# Cytomegalovirus, Epstein-Barr Virus, Herpes Simplex Virus, and Varicella Zoster Virus Infection Dynamics in People with Multiple Sclerosis from Northern Italy

**DOI:** 10.3390/pathogens13060499

**Published:** 2024-06-12

**Authors:** Peter A. Maple, Radu Tanasescu, Cris S. Constantinescu, Paola Valentino, Marco Capobianco, Silvia D’Orso, Giovanna Borsellino, Luca Battistini, Giovanni Ristori, Rosella Mechelli, Marco Salvetti, Bruno Gran

**Affiliations:** 1 Mental Health and Clinical Neuroscience Academic Unit, University of Nottingham School of Medicine, Nottingham NG7 2UH, UKbruno.gran@nottingham.ac.uk (B.G.); 2 Department of Neurology, Nottingham University Hospitals NHS Trust, Nottingham NG5 1PB, UK; 3 Cooper Neurological Institute, Cooper Medical School of Rowan University, Camden, NJ 08103, USA; 4 Neuroscience Institute, Cavalieri Ottolenghi, 10043 Orbassano, Italy; paolaval81@hotmail.com (P.V.);; 5 Department of Experimental Neuroscience, IRCSS Foundation Santa Lucia, 00179 Rome, Italy; 6 Department of Neurosciences, Mental Health and Sensory Organs, Centre for Experimental Neurological Therapies (CENTERS), Sapienza University of Rome, 00185 Rome, Italy; marco.salvetti@uniroma1.it; 7 Department of Human Science and Promotion of Quality of Life, San Raffaele Open University, 00166 Rome, Italy; 8 IRCCS, San Raffaele Roma, 00166 Rome, Italy; 9 Istituti di Ricovero e Cura a Carattere Scientifico (IRCCS) Istituto Neurologico Mediterraneo Neuromed, 86077 Pozzilli, Italy

**Keywords:** multiple sclerosis, Italy, human herpesviruses, Epstein–Barr virus, cytomegalovirus, herpes simplex virus, varicella zoster virus, seroprevalence, antibody levels

## Abstract

Previous exposure to Epstein–Barr virus (EBV) is strongly associated with the development of multiple sclerosis (MS). By contrast, past cytomegalovirus (CMV) infection may have no association, or be negatively associated with MS. This study aimed to investigate the associations of herpesvirus infections with MS in an Italian population. Serum samples (*n* = 200) from Italian people with multiple sclerosis (PwMS) classified as the relapsing-and-remitting clinical phenotype and (*n* = 137) healthy controls (HCs) were obtained from the CRESM Biobank, Orbassano, Italy. Both PwMS and HCs samples were selected according to age group (20–39 years, and 40 or more years) and sex. EBV virus capsid antigen (VCA) IgG, EBV nucleic acid-1 antigen (EBNA-1) IgG, CMV IgG, herpes simplex virus (HSV) IgG, and varicella zoster virus (VZV) IgG testing was undertaken using commercial ELISAs. EBV VCA IgG and EBNA-1 IgG seroprevalences were 100% in PwMS and 93.4% and 92.4%, respectively, in HCs. EBV VCA IgG and EBNA-1 IgG levels were higher (*p* < 0.001) in PwMS compared with HCs. For PwMS, the EBNA-1 IgG levels decreased with age, particularly in females. The CMV IgG seroprevalence was 58.7% in PwMS and 62.9% in HCs. CMV IgG seroprevalence increased with age. The HSV IgG seroprevalence was 71.2% in PwMS and 70.8% in HCs. HSV IgG levels were lower (*p* = 0.0005) in PwMS compared with HCs. VZV IgG seroprevalence was 97.5% in PwMS and 98.5% in HCs. In the population studied, several herpesvirus infections markers may have been influenced by the age and sex of the groups studied. The lack of a negative association of MS with CMV infection, and the observation of lower levels of HSV IgG in PwMS compared with HCs are findings worthy of further investigation.

## 1. Introduction

Multiple sclerosis (MS) is a leading cause of non-traumatic disability in young adults with a recently estimated 2.8 million people living with the disease worldwide [1]. According to the most recent Atlas of MS survey [2], the prevalence of MS in Italy is estimated at >200 per 100,000 people, notwithstanding regional variations [3]. The reasons why countries such as Italy have high population prevalences of MS remain to be determined.

MS is a chronic inflammatory, demyelinating disease of the central nervous system. Demyelination is a result of host autoimmunity and both genetic and environmental factors influence disease susceptibility and progression [4]. The association between past exposure to Epstein–Barr virus (EBV) and MS strongly suggests causation [5], although the definitive mechanisms responsible remain to be determined [6]. Infection with other human herpesviruses may also be associated with the development of MS. Human herpesvirus 6A infection has been associated with an increased risk of developing MS [7]. There is also evidence that varicella zoster virus (VZV) infection may be linked with the development of MS in certain geographical areas [8]. In the case of cytomegalovirus (CMV) infection, it has been suggested that it may have a “protective” role against the development of MS and that MS disease progression/severity may be linked to CMV antibody levels [9].

Nine human herpesviruses have been described [10] including the herpes simplex viruses (HSV1 and HSV2), varicella zoster virus (VZV), cytomegalovirus (CMV), Epstein–Barr virus (EBV), and human herpesviruses 6A and 6B (HHV6A and 6B). A common feature of infection with these viruses is that following primary infection a state of latency [11] is established with the host in which the virus remains dormant, with the capacity to reactivate at times when the host’s immune response is diminished. The human herpesviruses have been grouped into alphaherpesviruses (HSV 1 and 2 and VZV), betaherpesviruses (CMV, HHV6A, HHV6B, and HHV7), and gammaherpesviruses (EBV and Kaposi’s sarcoma herpesvirus) based on their cell tropisms and growth characteristics. The alphaherpesviruses are neurotropic [12], the betaherpesviruses (not including cytomegalovirus which infects several cell types) are lymphotropic [13], and the gammaherpesviruses are lymphotropic and oncogenic [14].

Infection with herpesviruses is common during childhood and, in the case of CMV, high childhood infection rates have been associated with several population indices including lower socioeconomic status [15]. Past infection with human herpesviruses can be shown by the detection of specific IgG, and serosurveys provide a useful means of estimating population trends in infection. In addition, the determination of antibody levels in populations may inform trends in immune responses and/or differences in the pathogenicity of the infecting viruses which are inapparent at an individual level. In this study, we report the results of sero-surveys of several human herpesviruses that have been conducted using serum samples obtained from Italian people with MS (PwMS) and HCs from northwestern Italy. The sera were collected under carefully standardized conditions from PwMS not currently undergoing treatment. Our aim was to assess virus exposures with associations to the development of MS in a northern Italian population using validated laboratory assays. Furthermore, we wished to assess the influence of basic parameters (age and sex) on seroprevalence and antibody levels which may be particularly relevant in the context of CMV infection.

## 2. Methods

### 2.1. Serum Samples

Serum samples from Italian PwMS (*n* = 200) and healthy controls (*n* = 137) were obtained from the CRESM Biobank (Centro Riferimento Regionale Sclerosi Multipla, AOU San Luigi Gonzaga, Orbassano, Italy), located at the Neuroscience Institute Cavalieri Ottolenghi (NICO), Orbassano, Italy. They were selected according to clinical status (relapsing-and-remitting phenotype, and not receiving disease-modifying immunotherapies), age group (20–39 years, and 40 or more years), and sex (see Table 1). The serum samples were stored at −80 °C. Serum samples were obtained from primary blood samples collected in serum tubes (BD Vacutainer, Becton, Dickinson and Company, Franklin Lakes, NJ, USA) and processed within two hours of collection according to CRESM Biobank standard procedures and international guidelines [16]. Blood samples were centrifuged at 3000× *g* 10 min, and serum supernatant was stored in the CRESM biobank at −80 °C in coded aliquots, until analysis, to avoid repeated freeze−thaw cycles.

### 2.2. Determination of Antibody Levels

Herpesviruses antibody levels were determined by commercial enzyme-linked immunosorbent assays supplied by Institut Virion\Serion, Würzburg, Germany. The following kits were used: Serion ELISA *classic* Epstein–Barr Virus Capsid Antigen (VCA) IgG ESR1361G, Serion ELISA *classic* Epstein–Barr Virus Nuclear Antigen-1 (EBNA-1) IgG ESR1362G, Serion ELISA *classic* Cytomegalovirus IgG ESR109G, Serion ELISA *classic* Varicella Zoster Virus IgG ESR104G, and Serion ELISA *classic* Herpes Simplex Virus 1/2 IgG ESR105G. The methodology has been described elsewhere [17]. Briefly, serum samples were diluted 1:100 in assay buffer and 100 uL was loaded into designated wells of antigen-coated microtiter plates together with calibrators, negative controls, and internal quality-control sera (Institut Virion\Serion, Würzburg, Germany Institut Virion). The plates were incubated for 60 min at 37 °C and then washed three times using an automated plate washer. Conjugate was added and the plates incubated for 30 min at 37 °C, and, after washing, substrate was added. The reaction was stopped after 30 min and the plates were read at 405 nm (reference 650 nm) using a microplate reader (BioRad Benchmark Plus, Hercules, CA, USA). Antibody levels were determined following interpolation of corrected optical density (OD) values from the manufacturer-supplied, lot-specific, standard curves. Significant numbers of sera when tested for EBV VCA IgG, and VZV IgG yielded ODs greater than the highest ODs of the linear portions of the respective standard curves. Because of sample volume limitations, these sera were not further tested at higher dilutions and they were given an assigned concentration following modelling beyond the highest concentration of antibody covered by the standard curve.

### 2.3. Statistics

Data were analysed using GraphPad Prism (GraphPad Prism version 9, GraphPad Software, Boston, MA, USA). The level of significance for statistical analyses was set at *p* < 0.05 for protocol-planned analyses, and *p* < 0.01 for unplanned (http://doi.org/10.17639/nott.7249, accessed on 9 June 2024). Associations of categorical data (e.g., sero-prevalence) were tested using Fisher’s exact test. The Mann–Whitney U test was used to evaluate associations of independent samples. Sample size estimates were not undertaken because several parameters were under investigation, for which previous quantitative estimates were not available.

## 3. Results

The PwMS male and female groups aged 18–39 years, and 40 years or more had similar age profiles as did the HCs except the HC males aged 40–77 years group which comprised only 15 samples (Table 1). Specific group comparisons were undertaken for all groups except the HC males aged 40–77 years group which comprised too few samples (Table 1). Quantitative antibody levels were compared by the calculation of geometric mean titres (GMT) as shown in the figures and tables.

### 3.1. Cytomegalovirus CMV IgG Seroprevalence and Antibody Levels in PwMS and Healthy Controls

**Figure 1:**
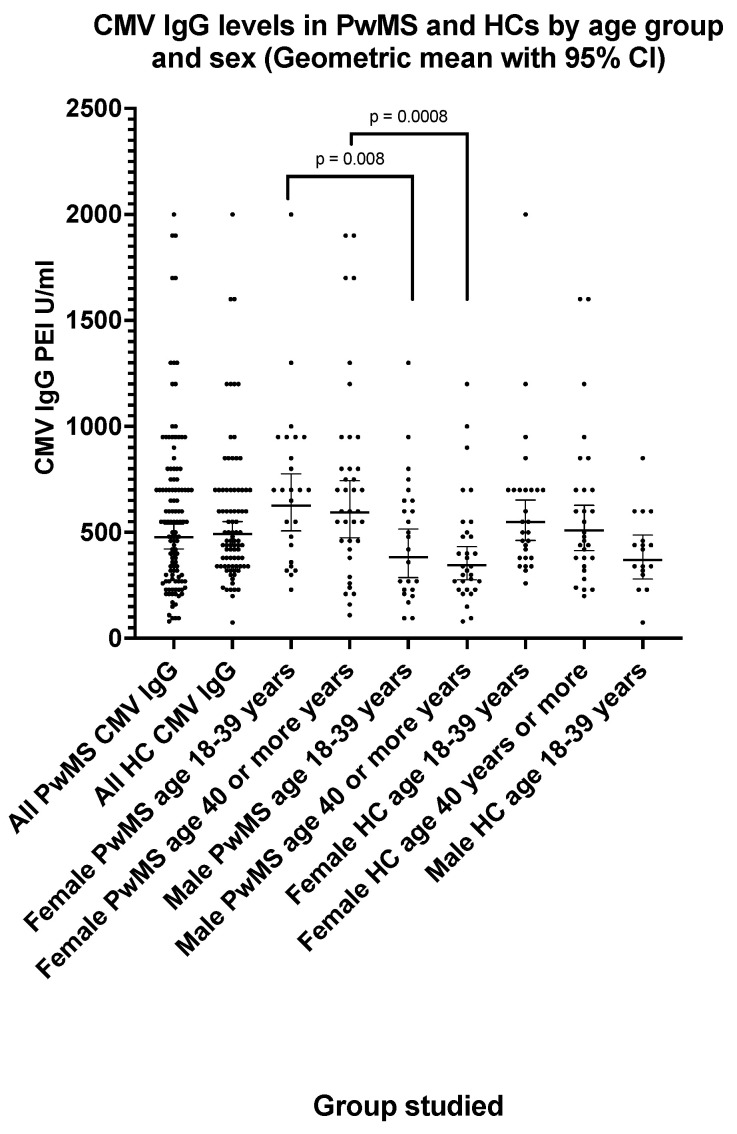
CMV IgG levels in PwMS and HCs by age group and sex (geometric mean with 95% confidence interval). The *p* values shown (two-tailed) were generated by the Mann–Whitney U test.

The CMV IgG seroprevalence (Table 2) was 58.7% (one equivocal result excluded) in PwMS and 62.9% in HCs (two equivocal results excluded). The CMV IgG seroprevalence increased with age in PwMS from 49.4% in those aged 18–39 years to 68.0% in those aged 40 years or more (Table 2). There was no significant difference (*p* = 0.95) between CMV IgG levels (Figure 1) in PwMS (GMT = 477 PEI U/mL; 95% CI: 422, 539) and HCs (GMT = 492 PEI U/mL; 95% CI 440, 550). The CMV IgG levels (Figure 1) were significantly higher (*p* < 0.01) in female PwMS compared with male PwMS (Figure 1). The CMV IgG level (GMT) in female PwMS aged 18–39 years was 627 PEI U/mL (95% CI: 507, 776) compared with 384 PEI U/mL (95% CI: 286, 515) in male PwMS of the same age group. Higher CMV IgG levels (*p* = 0.014) were also observed in female HCs aged 18–39 years (GMT = 549 PEI U/mL; 95% CI: 462, 652) compared with male HCs (GMT = 370 PEI U/mL; 95% CI: 280, 488). CMV IgG levels did not differ significantly between age groups.

### 3.2. Epstein–Barr Virus VCA IgG Seroprevalence and Antibody Levels in PwMS and Healthy Controls

**Figure 2:**
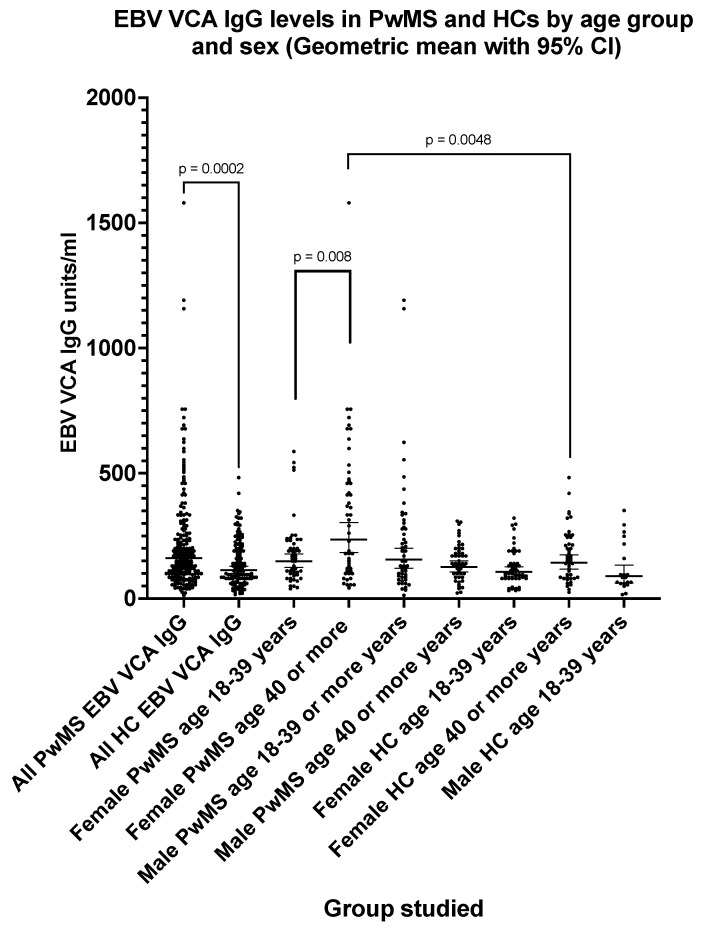
EBV VCA IgG levels in PwMS and HCs by age group and sex (geometric mean with 95% confidence interval). The *p* values shown (two-tailed) were generated by the Mann–Whitney U test.

The EBV VCA IgG seroprevalence (Table 3) was 100% (one equivocal result excluded) in PwMS and 93.4% in HCs. The EBV VCA IgG levels (Figure 2) were significantly higher (*p* = 0.0002) in PwMS (GMT = 162 EU/mL; 95% CI: 145, 181) compared with HCs (GMT = 114 EU/mL; 95%CI: 100, 129). The VCA IgG levels (Figure 2) were significantly higher (*p* = 0.008) in female PwMS aged 40 or more years (GMT = 235 EU/mL; 95% CI: 183, 303) when compared with female PwMS aged 18–39 years (GMT = 149 EU/mL; 95% CI: 124, 178). A similar age-related effect was not seen with male PwMS.

### 3.3. Epstein–Barr Virus EBNA-1 IgG Seroprevalence and Antibody Levels in PwMS and Healthy Controls

**Figure 3:**
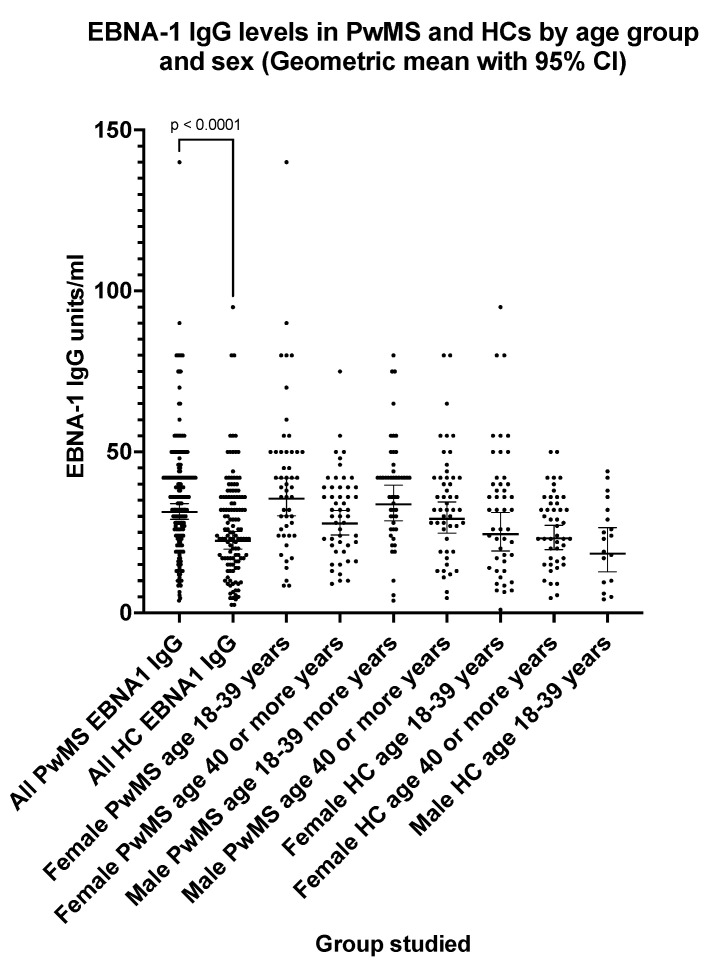
EBNA-1 IgG levels in PwMS and HCs by age group and sex (geometric mean with 95% confidence interval). The *p* value shown (two-tailed) was generated by the Mann–Whitney U test.

The EBNA-1 IgG seroprevalence (Table 4) was 100% (one equivocal result excluded) in PwMS and 92.4% in HCs (four equivocal results excluded). The EBNA-1 IgG levels (Figure 3) were significantly higher (*p* < 0.0001) in PwMS (GMT = 31.4 EU/mL; 95% CI: 29.0, 33.9) compared with HCs (GMT = 22.4 EU/mL; 95% CI: 19.8, 25.3). For PwMS (Figure 3), the EBNA-1 IgG levels did not differ significantly with sex; however, levels decreased with age particularly in females (*p* = 0.01). The EBNA-1 IgG GMT level for female PwMS aged 18–39 years was 35.5 EU/mL (95% CI: 30.2, 41.9) compared with 27.8 EU/mL (95% CI: 24.3, 31.8) in female PwMS aged 40 or more years.

### 3.4. Herpes Simplex Virus HSV IgG Seroprevalence and Antibody Levels in PwMS and Healthy Controls

**Figure 4:**
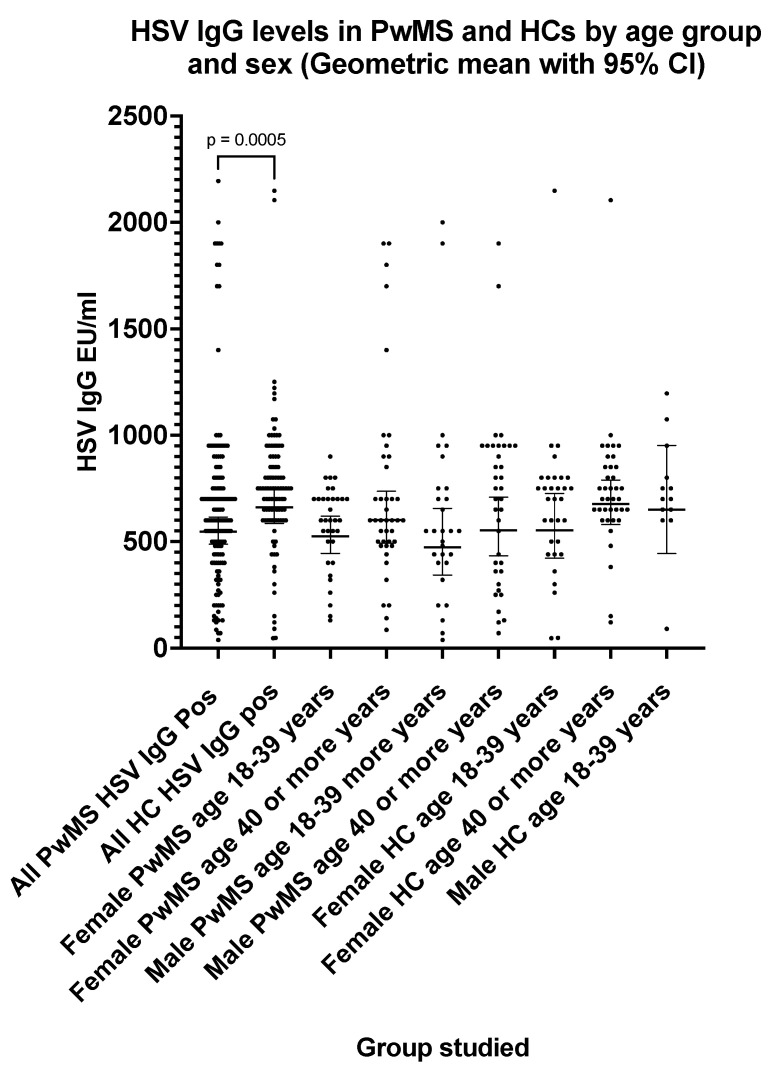
HSV IgG levels in PwMS and HCs by age group and sex (geometric mean with 95% confidence interval). The *p* value shown (two-tailed) was generated by the Mann–Whitney U test.

The HSV IgG seroprevalence (Table 5) was 71.2% in PwMS (two equivocal results excluded) and 70.8% in HCs (three equivocal results excluded). The HSV IgG levels were significantly lower (*p* = 0.0005) in PwMS compared with HCs (Figure 4). The HSV IgG GMT was 548 EU/mL (95% CI: 488, 615) in PwMS compared with 661 EU/mL (95% CI: 585, 748) in HCs (Table 5). The HSV IgG seroprevalence increased significantly (*p* = 0.01) with age in PwMS (Table 5); however, there was no significant difference in HSV IgG levels with age in either PwMS or HC groups.

### 3.5. Varicella Zoster VZV IgG Seroprevalence and Antibody Levels in PwMS and Healthy Controls

**Figure 5:**
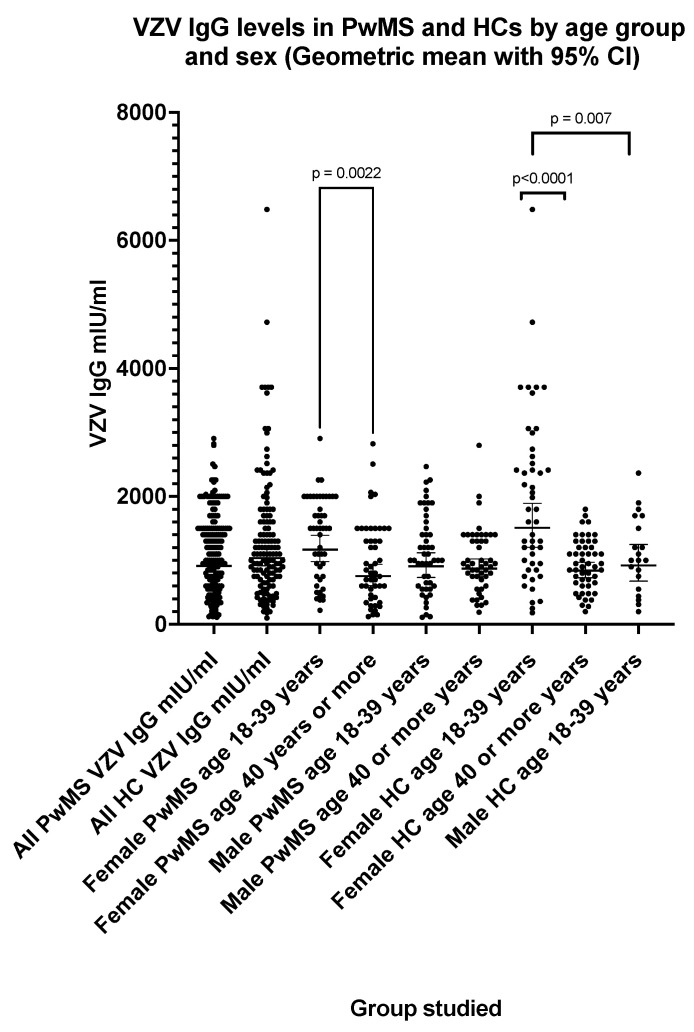
VZV IgG levels in PwMS and HCs by age group and sex (geometric mean with 95% confidence interval). The *p* values shown (two-tailed) were generated by the Mann–Whitney U test.

The VZV IgG seroprevalence (Table 6) was 97.5% in PwMS and 98.5% in HCs (three equivocal results excluded). There was no significant difference (*p* = 0.25) between VZV IgG levels (Table 6) in PwMS (GMT = 913 mIU/mL; 95% CI: 828, 1006) and HCs (GMT = 1035 mIU/mL; 95% CI: 914, 1173). The VZV IgG levels significantly decreased with age between those aged 18–39 years and those aged 40 or more years (Figure 5) both in female PwMS (*p* = 0.002) and female HCs (*p* < 0.0001). The VZV IgG GMT was 1168 mIU/mL (95% CI: 980, 1391) in female PwMS aged 18–39 years and 752 mIU/mL (95% CI: 601, 939) in female PwMS aged 40 or more years. Higher VZV IgG levels (*p* = 0.007) were observed in female HCs aged 18–39 years (GMT = 1507 mIU/mL; 95% CI: 1203, 1889) compared with male HCs aged 18–39 years (GMT = 918 mIU/mL; 95% CI: 675, 1249); however, a significant difference (*p* = 0.053) was not observed between the same PwMS groups (Figure 5).

### 3.6. Burden of Herpesviruses Infections in PwMS and Healthy Controls

The burden of herpesviruses infections in the PwMS and HC populations was similar (Table 7).

## 4. Discussion

It is now widely recognized that EBV infection is an essential precursor for the development of MS. The presence of EBV VCA IgG and EBNA-1 IgG which, taken together, are indicative of past EBV infection [18] for all Italian PwMS, supports the fundamental role of EBV infection in the development of MS [19,20]. Furthermore, it has also been reported [21,22] that EBV VCA IgG and EBNA-1 IgG levels are higher in PwMS compared with controls and this trend has also been seen in our study population of PwMS from northwest Italy. It is important to gather regional data because even within national boundaries the prevalence of MS can vary widely [3]. In the context of MS, various findings have been reported for CMV IgG seroprevalence compared to controls in PwMS. There have been several reports that CMV seroprevalence is lower in PwMS compared with non-MS controls: for example, from Scandinavia [23], Spain [24], the USA [25], and the UK [17]. Yet in other studies, a negative association of CMV infection with MS has not been observed [26,27] and there is some evidence to suggest that these differences may be linked to regional and ethnic-group-related factors [28,29]. A greater understanding of how CMV infection potentially protects against MS is needed. This was highlighted by a recent study by Vietzen and colleagues [30] who reported a potential interaction between human genetic predisposition and past CMV infection influencing protective immune responses against autoimmune cells and defining the individual risk for MS. Previously, it has been shown that UL40-signal-peptide variants encoded by CMV are involved in NK-cell evasion pathways [31].

VZV IgG seroprevalence rates were similar in PwMS and healthy controls and a lack of an association of VZV infection with MS has been noted in several studies [32,33]. Conversely, other studies [34,35] have reported an association of a history of VZV infection with MS, and a recent meta-analysis [36] has suggested geographic heterogeneity in the association of VZV IgG seropositivity with MS. We have also assessed HSV seropositivity in PwMC and HCs. HSV1 and HSV2 are antigenically very similar and difficult to differentiate by serological assays [37,38]. In our study, an assay was used that detects antibodies to both HSV1 and HSV2, and total HSV IgG seroprevalences of approximately 70% were found for both PwMS and HCs. An interesting observation, worthy of further investigation, was that total HSV IgG levels were significantly lower (*p* = 0.0005) in PwMS compared with HCs. Several epidemiological, clinical, and animal studies [39] have supported the involvement of HSV-1 in MS; however, there is considerable evidence that HSV infection is not associated with MS [40,41].

Multiple sclerosis is more prevalent in females than males and typically manifests during early adulthood [42]. Comparisons of herpesvirus antibody levels and seroprevalences by sex in our study revealed significant differences between females and males for CMV IgG. Sex, age, and CMV infection are all known to influence immune function [43,44]. Few studies of herpesvirus infections in PwMS have specifically addressed these issues. In our study, higher CMV IgG levels were observed in both female PwMS and HCs compared with their male counterparts. In the context of MS [45], high CMV IgG levels have been associated with a more benign MS disease course, so our findings merit further investigation at a clinical level. It has also been shown [46,47] that CMV infection influences the host immune response to EBV. Both beta and gamma human herpesviruses possess extensive immunomodulatory capacities and co-infection can manifest both agonistic and antagonistic interactions with the host immune system [48] The Italian population studied showed a high seroprevalence (>95%) of VZV IgG in all the groups studied, which is in accordance with high VZV IgG seroprevalences reported for adults from several Western European countries [49,50]. In our study, VZV IgG levels in female adults significantly decreased with age, and a general decline in VZV IgG levels in Italian adults has been noted in other studies [51].

Multiple sclerosis is a disease resulting from genetic predisposition and the influence of environmental factors. Previous infection with EBV is considered a pre-requisite for subsequent MS and the data from our study further support this view. Infection by CMV may have a protective effect, at least in some populations. VZV infection may be associated with the development of MS particularly in non-European regions with different virus transmission characteristics [52]. There is strong evidence [7,53] that another human herpesvirus—HHV6A—is also associated with the development of MS. We plan to investigate this association in a separate study. The events that combine for MS to develop may occur many years before the appearance of disease. There is a need to fully characterize herpesviruses infection dynamics in multiple sclerosis populations as they can have significant impacts on immune responses, which are also influenced by age and sex [54].

In conclusion, we were motivated to undertake a careful virological assessment of a well characterized population from northern Italy for several reasons. Firstly, we wished to provide high quality serological data for future sero–epidemiological studies. When undertaking serological testing it is essential to use validated assays, quality assure the results, and use sera of known provenance. In our study, commercial assays were used, and standard curve interpolations were undertaken using lot-specific standard curves supplied by the manufacturer. Furthermore, assay run-to-run variation was monitored using dedicated internal quality control sera. Finally, the sera tested had been collected and stored under defined and standardized conditions from a defined population of PwMS (RRMS) who were not currently undergoing treatment. Our second objective was to assess the impact of age and sex on herpesvirus antibody profiles. The categorization of our populations into those aged under 40 years and those aged 40 years and over was motivated by the observations of Alari-Pahissa et al. [24] who reported a significant difference in CMV seroprevalence in PwMS age 40 years or under (45%) compared with controls (67%) of a similar age. This effect was not apparent in those aged over 40 years.

Notwithstanding the provision of seroprevalence and herpesviruses antibody levels data for PwMS in northern Italy, noteworthy findings from our study, using this highly standardized approach to laboratory testing, include the observation of no apparent protective effect of past CMV infection in PwMS against the development of MS. Additionally, we have shown HSV IgG levels to be significantly lower in PwMS which is a finding worthy of further investigation. Finally, although not novel in itself, we have confirmed several herpesvirus antibody associations with sex and age which may inform the appropriate selection of population panels when contemplating seroepidemiological studies.

## Figures and Tables

**Table 1:** Descriptive statistics of Italian PwMS (all clinically designated relapsing-and-remitting phenotype) and healthy controls: totals and by age and sex groups selected.

Group Tested/Descriptive Statistic	Total Samples for All Groups		Females 18–39 Years		Males 18–39 Years		Females 40–77 Years		Males 40–77 Years	
	PwMS	HC	PwMS	HC	PwMS	HC	PwMS	HC	PwMS	HC
No. of samples	200	137	50	50	50	22	50	50	50	15
Median age	39.5	38	28.5	27.5	30	28	46	48	47	57
Mean age	38.6	39.3	29.4	28.8	30	28.6	47	49.2	48	57.1
Mean std. dev.	10.8	13	6.58	4.88	5.75	4.98	5.65	6.81	6.53	10.3
Lower 95% CI	37.1	37.1	27.6	27.4	28.4	26.4	45.4	47.3	46.2	51.4
Upper 95% CI	40.1	41.5	31.3	30.2	31.7	30.8	48.6	51.1	49.9	62.8

PwMS = people with multiple sclerosis, HC = healthy control, std. dev. = standard deviation, lower 95% CI = lower 95% confidence interval of mean, upper 95% CI = upper 95% confidence interval of mean.

**Table 2:** CMV IgG parameters in Italian MS Biobank MS sera and healthy control sera.

Parameter	MS Sera (*n* = 200)	Healthy Controls (*n* = 137)
Seroprevalence (overall)	58.7% (*n* = 199) *	62.9% (*n* = 135) **
Seroprevalence (females)	62.6% (*n* = 99) *	58.1% (*n* = 98) **
Seroprevalence (males)	55.0% (*n* = 100)	Insufficient samples
Seroprevalence (<40 years)	49.4% (*n* = 99) *	56.0% (females only) *n* = 50
Seroprevalence (≥40 years)	68.0% (*n* = 100)	60.4% (females only) *n* = 48 **
GMT EU/mL (overall) lower/upper 95% CI	477 PEI-U/mL * (422, 539)	492 PEI-U/mL ** (440, 550)
Median EU/mL (overall) 25th/75th percentile	550 PEI U/mL * (288, 750)	460 PEI-U/mL ** (340, 700)

* 1 equivocal excluded, ** 2 equivocals excluded, CI = confidence interval.

**Table 3:** EBV VCA IgG parameters in Italian MS Biobank MS and healthy control sera.

Parameter	MS Sera (*n* = 200)	Healthy Controls (*n* = 137)
Seroprevalence (overall)	100% (1 equivocal) (*n* = 199) *	93.4% (*n* = 137)
Seroprevalence (females)	100% (*n* = 100)	94.0% (*n* = 100)
Seroprevalence (males)	100% (1 equivocal) (*n* = 99) *	Insufficient samples
Seroprevalence (<40 years)	100% (1 equivocal) (*n* = 99)	96.0% (females only) (*n* = 50)
Seroprevalence (≥40 years)	100% (*n* = 100)	92.0% (females only) (*n* = 50)
GMT EU/mL (overall) lower/upper 95% CI	162 EU/mL * (145, 181)	114 EU/mL (100, 129)
Median EU/mL (overall) 25th/75th percentile	165 EU/mL * (100, 261)	115 EU/mL (80.0, 200)

* Equivocal sample not included, CI = confidence interval.

**Table 4:** EBNA-1 IgG parameters in Italian MS Biobank MS sera and healthy control sera.

Parameter	MS Sera (*n* = 200)	Healthy Controls (*n* = 137)
Seroprevalence (overall)	100% (*n* = 199) *	92.4% (*n* = 133) ****
Seroprevalence (females)	100% (*n* = 100)	93.8% (*n* = 98) **
Seroprevalence (males)	100% (*n* = 99) *	88.5% ** (*n* = 35)
Seroprevalence (<40 years)	100% (*n* = 99) *	93.8% (1 equivocal) (females only) *n* = 49 *
Seroprevalence (≥40 years)	100% (*n* = 100)	93.8% (1 equivocal) (females only) *n* = 49 *
GMT EU/mL (overall) lower/upper 95% CI	31.4 U/mL * (29.0, 33.9)	22.4 EU/mL **** (19.8, 25.3)
Median EU/mL (overall) 25th/75th percentile	36.0 U/mL * (24.0, 42.0)	25.0 EU/mL **** (17.0, 36.0)

* 1 equivocal excluded, ** 2 equivocals excluded, **** 4 equivocals excluded, CI = confidence interval.

**Table 5:** HSV IgG parameters in Italian MS Biobank MS sera and healthy control sera.

Parameter	MS Sera (*n* = 200)	Healthy Controls (*n* = 137)
Seroprevalence (overall)	71.2% (*n* = 198) **	70.8% (*n* = 134) ***
Seroprevalence (females)	76.0% (*n* = 100)	72.1% (*n* = 97) ***
Seroprevalence (males)	66.3% (*n* = 98) **	Insufficient samples
Seroprevalence (<40 years)	63.6% (*n* = 99) *	66.6% (females only, *n* = 48) **
Seroprevalence (≥40 years)	78.7% (*n* = 99) *	79.5% (females only, *n* = 49) *
GMT EU/mL (overall) lower/upper 95% CI	548 EU/mL ** (488, 615)	661 EU/mL *** (585, 748)
Median EU/mL (overall) 25th/75th percentile	600 EU/mL ** (440, 800)	750 EU/mL *** (600, 900)

* 1 equivocal excluded, ** 2 equivocals excluded, *** 3 equivocals excluded, CI = confidence interval.

**Table 6:** VZV IgG parameters in Italian MS Biobank MS sera and healthy control sera.

Parameter	MS Sera (*n* = 200)	Healthy Controls (*n* = 137)
Seroprevalence (overall)	97.5% (*n* = 200)	98.5% (*n* = 134) ***
Seroprevalence (females)	98.0% (*n* = 100)	98.9% (*n* = 99) *
Seroprevalence (males)	97.0% (*n* = 100)	Insufficient samples
Seroprevalence (<40 years)	98.0% (*n* = 100)	97.9% (females only) *n* = 49 *
Seroprevalence (≥40 years)	97.0% (*n* = 100)	100% (females only) *n* = 50
GMT EU/mL (overall) lower/upper 95%CI	913 mIU/mL (828, 1006)	1035 mIU/mL *** (914, 1173)
Median EU/mL (overall) 25th/75th percentile	1000 mIU/mL (600, 1500)	1100 mIU/mL *** (700, 1650)

* 1 equivocal excluded, *** 3 equivocals excluded, CI = confidence interval.

**Table 7:** Comparison of burden of herpesviruses infections in PwMS and healthy controls.

Parameter	MS Sera (*n* = 200)	Healthy Controls (*n* = 137)
1 infection marker only	0%	2.18%
2 infection markers only	1.0%	2.18%
3 infection markers only	16.5%	18.2%
4 infection markers only	37.5%	35.7%
5 infection markers only	45.0%	41.6%

The infection markers were EBV VCA IgG, EBNA-1 IgG, CMV IgG, HSV IgG and VZV IgG. Equivocal results were treated as negatives.

## Data Availability

The data presented in this study are available on request from the corresponding author (P.A.M.) and senior author (B.G.). The data are not publicly available due to GDPR regulations on research subjects (patients and healthy controls).
